# Long-term conditions and symptom-based disorders – A community perspective

**DOI:** 10.1016/j.fhj.2026.100499

**Published:** 2026-01-09

**Authors:** Chris Barker

**Affiliations:** Mersey Care NHS Foundation Trust, Community Pain Service, V7 Building, Kings Business Park, Prescot, Merseyside, L34 1PJ, UK

**Keywords:** Long-term conditions, Pain, Functional neurological disorder, Symptom based disorder, Community services

## Abstract

Long-term conditions (LTCs) and symptom-based disorders (SBDs), such as chronic primary pain and functional neurological disorders, challenge traditional healthcare models. This article explores the facets important in the clinical management of SBDs through a community lens, advocating for integrated, person-centred approaches that move beyond the biomedical paradigm. It examines how we conceptualise and communicate about SBDs, the need for holistic assessment and management strategies, and limitations of current commissioning frameworks. The role of peer support and co-constructed networks is also highlighted drawing on peer-reviewed literature, policy and real-world service innovations. The article also acknowledges the political landscape surrounding the NHS, noting the tension between short-term political cycles and the need for long-term strategic planning. It calls for a reimagining of care that prioritises function, agency and collaboration across sectors, and emphasises the importance of integrated care, shared decision-making and governance that supports sustainable, community-embedded solutions for people living with complex, chronic symptoms.

## Introduction

The rising burden of long-term conditions (LTCs) and symptom-based disorders is a growing challenge to health systems. These conditions often span physical, psychological and social domains, yet our models of care remain rooted in a biomedical paradigm that prioritises diagnosis, pathology and morbidity over understanding symptoms, lifestyle optimisation and recovery. It could be argued that in the UK, we have become a ‘National Illness Service’ as opposed to the National Health Service we aspire to.^1^^,^^2^

Medicalised approaches, while essential in acute care, frequently fall short in addressing the complexity and chronicity of LTCs. Often the focus is upon minimising symptoms without considering the enablement of wellbeing, overlooking the role of lifestyle, behaviour and community in shaping outcomes.^3^ The iatrogenic harm brought through fragmented care, repeated investigations and often unhelpful biomedical narratives result in disempowered patients who feel confused, unheard, ill-equipped and poorly supported to navigate an overly complex system.^4^

Integrative approaches such as lifestyle medicine aim to acknowledge the multifactorial nature of LTCs and consider the ‘whole person’ and also their role in maintaining health. A collaborative expert–expert ethos exists, where both clinician and patient are considered experts. This collaborative approach aims to combine the best from both individuals, resulting in a co-constructed, truly person-centred management strategy. It is the additional role of the clinician therefore to be an enabler, with experience to recognise how and when to focus upon a patient’s own skills.^5^^,^^6^

Community-based services are often in a good place to offer evidence-based rehabilitative approaches in LTCs. They can bring together a multiprofessional team to support people living with persistent symptoms through education, shared decision-making and goal-oriented rehabilitation. Prioritisation of building a clear shared narrative fosters autonomy and builds capacity for self-management. Peer support and experts by experience can more easily be embedded into this approach, helping to normalise recovery and reduce stigma.^7^^,^^8^

This model, however, challenges traditional commissioning frameworks by focusing on individualised outcomes, rather than conventional key performance indicators or generic wellness outcomes. It also highlights the need for integrated services across primary, secondary and community care, and for investment in community-led initiatives that can complement NHS provision.^9^^,^^10^ Reimagining care for LTCs means moving beyond the biomedical model to one that embraces complexity, values lived experience, and supports recovery in the context of people’s lives.

## Understanding symptom-based disorders – how we think about and talk about them

Symptom-based disorders (SBDs) such as fibromyalgia, functional neurological disorder (FND), irritable bowel syndrome and chronic fatigue syndrome present a significant challenge to traditional models of healthcare delivery. These conditions are characterised by persistent symptoms – pain, fatigue, sensory, motor or cognitive dysfunction – that are not fully explained by identifiable structural pathology. Estimates suggest that up to 30% of LTCs are accounted for by SBDs, and similarly, up to 30% of primary care consultations involve symptoms without a clear organic cause.^11^ This has substantial implications for healthcare utilisation and patient experience.

Despite their prevalence, SBDs remain poorly understood. They are not singular entities but reflect a convergence of biological dysregulation, psychological factors and social context.^12^ Neuroimaging studies in FND and chronic pain syndromes reveal altered brain network activity, particularly in regions governing attention, emotion and sensorimotor integration.^13^ These changes are functional, representing neuroplasticity, and not degenerative. The nervous system is inherently adaptive; its calibration is influenced by environmental inputs, behavioural patterns and internal states. It is therefore plausible – and increasingly evidenced – that persistent symptoms may arise from maladaptive processing rather than fixed pathology. Importantly, these changes are modifiable, highlighting the potential for recovery through targeted rehabilitation and behavioural interventions.^13^

This distinction – dysregulation versus degeneration – is critical. Medical education has traditionally focused on disease models rooted in structural pathology. However, SBDs challenge this paradigm, requiring clinicians to engage with multifactorial systems involving stress physiology, sleep disruption, microbiome–host interactions and epigenetic influences.^12^^,^^14^

Understanding this dynamic reframes how we talk about these conditions. Language matters. Terms such as ‘degeneration’ imply irreversible decline, whereas ‘dysregulation’ suggests potential for recalibration and recovery. This shift supports a more hopeful, person-centred narrative and aligns with emerging rehabilitative models of neuroplasticity and functional optimisation. It also helps avoid the damaging overmedicalisation of distress, which can risk a perceived attribution of blame for symptoms.^15^ Training the body – through movement, graded activity and sensory retraining – is not merely symptomatic management, but a way to recalibrate neural circuits and restore function. This perspective encourages patients to engage actively in their recovery, supported by multidisciplinary teams that validate their experience and promote self-efficacy.

Wider LTCs (eg type 2 diabetes, ischaemic heart disease) also carry an individual element of SBD. The binary distinction between ‘disease’ and ‘non-disease’ is increasingly unhelpful in this context. A systems-based understanding – recognising the interplay between biological, psychological and social factors – is essential. Modifying lifestyle can improve overall wellness and wellbeing, but also inherently improve biological aspects of conditions (eg improvements in insulin resistance or cardiovascular fitness). Often, the key to initially engaging such lifestyle modifications lies in the complex 1:1 interaction in a consultation.

## Reimagining assessment and management for SBDS: beyond the biomedical model

The care of symptom-based disorders demands a fundamental reorientation of clinical thinking, service design and commissioning. The prevailing biomedical model – rooted in diagnosis, pathology and intervention – has delivered transformative advances in acute care. Yet, it falls short in addressing the complexity, chronicity and lived experience of LTCs and SBDs.^15^

Traditional assessments often exclude critical dimensions of health. Whole-person evaluations are rarely whole; they focus on identifying pathological cues while neglecting psychosocial factors, functional capacity and personal agency. Skills such as resilience, adaptability and motivation – central to recovery and wellbeing – are seldom measured or supported.^16^ A wider focus avoids a ‘see–treat–discharge’ model ill-suited to conditions that require continuity, collaboration and co-production. Holistic assessment also involves exploring motivation and readiness for change. Many patients with SBDs are not seeking a ‘quick fix’, but want to understand their symptoms and regain control. This can be facilitated by integrative approaches that span sectors, specialties and communities.

Integrated care models, particularly those involving multidisciplinary teams and community-based support, have shown promise in improving outcomes, reducing hospital admissions, and enhancing quality of life.^17^ Communication is central to holistic assessment. A collaborative ethos – where the clinician is an ‘invited expert’ into the patient’s life – helps build trust and shared understanding. Patients often have good reasons for their behaviours and beliefs, shaped by lived experience, trauma and cultural context. Shared decision-making frameworks emphasise the importance of mutual expertise and have been shown to improve outcomes in complex care settings.^18^

Holistic assessment requires a nuanced, person-centred approach that balances clinical vigilance with therapeutic curiosity. A foundation of holistic clinical assessment is the ability to identify and respond to ‘red flags’ – clinical indicators of serious pathology that require urgent investigation. This remains a core clinical responsibility. Many patients present with functional symptoms alongside comorbid medical conditions; understanding the balance between pathology and dysregulation is itself a clinical skill. Functional symptoms reflect altered neurobiological processing, and treating both pathology and dysregulation, when they coexist, requires intelligent, integrated care planning.^15^

Ultimately, holistic assessment is more of a relational process than a checklist. It requires curiosity, presence of mind, and a willingness to walk alongside patients on their journey. It also demands a recognition of our own limits as clinicians, resisting the temptation to be considered the ‘sole expert in the room’, and instead collaborating with the person as a co-expert. Truly listening to a person and understanding their hopes remains a vital approach; our own perception of their needs may be mistaken; *‘it’s a long day on the golf course when you don’t know where the hole is.’*

Outcome measures in this context are important. Validated tools often reflect what systems think wellness is, rather than what matters to patients. Many are pathology-focused, failing to capture motivation, energy, or readiness to change. Tools that combine individualised, wellness-oriented outcomes with scalable motivational support are rare, and validation remains a challenge.^19^

Examples of this, however, do exist. iGro is a user-defined scaling tool for measuring and motivating change across personalised dimensions. Developed originally within chronic pain rehabilitation,^20^ it allows the user to define five dimensions considered important to ‘grow’ as part of their recovery. This is distinct from factors often prioritised in validated tools that focus on what to ‘shrink’ (eg pain, anxiety). The user defines where they see themselves on a 0–10 scale for each dimension. The clinician and user then explore what the chosen number means (eg 2/10 = ‘I can walk 100 yards’ or another dimension might be: 5/10 = ‘I get around 4 h sleep per night’). This personalisation enables scaling to be applied: ‘You said you were 2/10 – what does 3/10 look like for you? How do you see yourself getting there?’. This multi-dimensional approach translates into both a truly person-centred outcome measure and a motivational tool, and lends itself to SBDs especially due to their diversity and individuality. Digitisation of such tools offers potential, though their intelligent integration into care remains uneven.^21^

## Meaningful support networks for symptom-based disorders

Simon Stevens, former chief executive of NHS England, recognised peer support as an ‘untapped resource’ in the NHS, advocating for its integration into care pathways and community partnerships.^22^ Patients also often learn best from others who share similar experiences. Peer support offers a unique form of empathy, relational learning, shared identity and lived expertise. Often peers connect with patients through sharing their own hardships and, importantly, *how they overcame them*.

Systematic reviews have shown that peer support can improve quality of life, self-efficacy, and engagement with services across a range of chronic conditions.^23^ Social identity theory supports this, suggesting that advice and encouragement from someone within the same experiential group is more likely to be accepted and internalised.^24^ Examples from long COVID also demonstrate that patients often turn to online and community-based peer groups to fill gaps in clinical care, share coping strategies, and to validate their experiences^25^ – *elements often missing in conventional healthcare encounters.*

Peer support can be effective when integrated within NHS clinical services. In this context, the service itself ‘home grows’ experts by experience (EBEs), integrating them into support roles. This approach allows effective support, governance and training for EBEs, while enabling multiple groups focusing on different aspects of wellbeing to flourish safely.

Building meaningful networks for SBDs requires investment in infrastructure – training for peer supporters, governance frameworks, and commissioning models that recognise their value and cost-effectiveness. Community services are sometimes commissioned with this type of support within their specification, but this is not yet commonplace

## Political and commissioning landscape

The NHS remains a political institution – electorally sensitive and structurally complex. Despite widespread recognition of the need for long-term strategic planning, political control over the NHS continues to be shaped by short-term electoral cycles. The BMJ Commission (2024) argues that the NHS is ‘beholden to politics’, with successive governments reluctant to relinquish control, even when cross-party consensus could offer greater stability and sustainability.^26^

This politicisation has consequences for service design and integration. The Major Conditions Strategy (DHSC, 2023) identifies persistent fragmentation between physical and mental health services, despite decades of policy rhetoric advocating for holistic care. The strategy calls for integrated models that reflect the realities of multimorbidity and the need for joined-up support across the life course.^27^

Community-led care faces similar challenges. The Neighbourhood Health Guidelines (NHS England, 2025) advocate for locally embedded, multidisciplinary teams and peer-led support. Yet funding mechanisms for grassroots initiatives remain limited, and commissioning rarely prioritises prevention or community education.^28^ Disinvestment in public health undermines efforts to build resilience and reduce demand on acute services. This shift also undermines efforts to reduce health inequalities and promote recovery. Evidence from The King’s Fund highlights the urgent need to reinvest in community education, early intervention and participatory models of care.^29^

Integrated care systems (ICSs) were introduced to address these gaps, yet their implementation has been uneven. The NIHR PRUComm literature review (2019) notes that while ICSs offer a framework for collaboration, organisational silos, variable governance and inconsistent commissioning practices continue to undermine their potential.^30^ As austerity tightens, it would seem that the implementation of more integrated care is likely to move further away

Digital transformation also outpaces delivery. NHS England’s Fit for the Future plan (2024) outlines a vision for IT systems that support personalised care, wellbeing tracking and proactive management. However, current infrastructure remains geared toward pathology measurement and risk mitigation, rather than enabling recovery, motivation or functional improvement.^31^

Ultimately, the political landscape shapes not only what is funded, but how health is conceptualised. A shift toward long-term, cross-sector strategy – grounded in population health, prevention and community participation – is urgently needed. This requires political courage, collaborative governance, and a willingness to invest in much longer-term systems that can function in the real world.

## Summary

Symptom-based disorders demand a shift in how we think, assess and commission care. The biomedical model, while foundational, is insufficient for addressing the complexity and chronicity of these conditions. A systems-based, person-centred approach – grounded in shared decision-making, functional rehabilitation and community engagement – is essential. Holistic assessment must balance clinical vigilance with therapeutic curiosity, recognising the interplay between pathology and dysregulation. Peer support networks, co-constructed with patients and communities, offer scalable, relational solutions that complement clinical care. However, political and structural barriers persist. Fragmentation across services, inadequate metrics and short-term policy cycles hinder progress. The NHS must embrace long-term, cross-party strategies that invest in prevention, integration and digital infrastructure designed to support wellbeing. Commissioning should reflect what matters to patients, valuing growth, participation and recovery. By reimagining care for SBDs through a community lens, we can build systems that are more inclusive, resilient, and responsive to the realities of living with symptom-based disorders.

## CRediT authorship contribution statement

**Chris Barker:** Writing – review & editing, Writing – original draft, Visualization, Conceptualization.

## Declaration of competing interest

The authors declare the following financial interests/personal relationships which may be considered as potential competing interests: I have been paid by NICE for consultancy work involving a pharmaceutical company in the last 3 years. If there are other authors, they declare that they have no known competing financial interests or personal relationships that could have appeared to influence the work reported in this paper.
